# Vicarious trauma in Child and adolescent psychiatry residents in Tunisia

**DOI:** 10.1192/j.eurpsy.2025.1010

**Published:** 2025-08-26

**Authors:** G. Amira, S. Bourgou, F. Charfi

**Affiliations:** 1 Child psychiatry Department, Mongi Slim Hospital, La Marsa, Tunisia

## Abstract

**Introduction:**

Vicarious trauma can significantly affect the physical and mental health of mental health professionals, as well as their ability to provide quality of care, particularly in the population of child and adolescent psychiatry residents who frequently encounter traumatic narratives.

**Objectives:**

This study aims to determine the prevalence of vicarious trauma in child and adolescent psychiatry residents.

**Methods:**

This is a cross-sectional study conducted from 19 august to 16 september 2024. An anonymous Google Forms questionnaire was sent to child psychiatry residents via the college email and private groups. We used the Compassion Fatigue Questionnaire to measure levels of vicarious trauma.

**Results:**

A total of 48 child psychiatry resident out of 70 participated in the study. The sex ratio was 1:8,6. The mean age was 28,8 years. The residents were married in 33,4% of cases, in a romantic relationship in 29,1% and single in 37,5%. We found that 54,1% of residents practiced a leisure activity, 22,9% had history of psychiatric disorder and 14,5% had addictive behaviours. We found that 74% of the residents exhibited a high to very high risk of vicarious trauma. The mean score of Compassion Fatigue Questionnaire was 44,18.

**Conclusions:**

This study underscores the need to recognize and address vicarious trauma among child and adolescent psychiatry residents in Tunisia, as its effects can detrimentally influence both clinician well-being and patient care. By fostering an awareness of this often-overlooked issue and integrating supportive measures into residency training, it can help residents develop healthier coping strategies and resilience.

**Disclosure of Interest:**

None Declared

